# Chemical composition, determination, and prediction of energy values of different sugarcane yeasts for broiler chickens

**DOI:** 10.1016/j.vas.2026.100679

**Published:** 2026-05-05

**Authors:** Emanuela Nataly Ribeiro Barbosa, Carlos Bôa-Viagem Rabello, Marcos Jose Batista dos Santos, Claudia Costa Lopes, Edney Pereira Silva, Júlio Cezar dos Santos Nascimento, Camilla Mendonça Silva, Lilian Francisco Arantes de Souza, Lucas Rannier Ribeiro Antonino Carvalho, Maria do Carmo Mohaupt Marques Ludke

**Affiliations:** a Departamento de Ciência Animal, Universidade Federal Rural de Pernambuco, 52171-900, Recife PE, Brazil; b Escola de Agricultura de Jundiaí, Universidade Federal do Rio Grande do Norte, 59280-000, Macaíba RN, Brazil; c Departamento de Zootecnia, Universidade Estadual Paulista, 14884-900, Jaboticabal SP, Brazil; d Department of Physiology and Pharmacology, Karolinska Institutet 171 77, Stockholm, Sweden

**Keywords:** Digestibility, Metabolizable energy, Regression equations, *Saccharomyces cerevisae*

## Abstract

The growing demand for sustainable and cost-effective feed ingredients in poultry production has highlighted agro-industrial by-products like sugarcane yeast, which can address environmental waste from the sugar and alcohol industry while providing nutritional benefits for broiler chickens. This study aimed to determine the energy values of 4 yeast samples and develop prediction equations based on their chemical composition, hypothesizing that variations would influence metabolizable energy across broiler growth stages. Samples were analyzed for physicochemical properties (e.g., crude protein, CP: 14.94–21.12%; gross energy: 4011–4268 kcal kg^-1^), and digestibility tests were conducted on 690 male Cobb 500 broilers in a completely randomized design across 4 phases (1–8 days, 11–18 days, 21–28 days, and 31–38 days), using reference and yeast-enriched diets (30% inclusion), followed by multiple regression analysis. Apparent metabolizable energy (AME) and corrected AME (AME_n_) values ranged from 800 to 2223 kcal kg^-1^ and 698–2069 kcal kg^-1^, respectively, with the best equation being AME = 8905.51 – 32.1006 × age – 81.048 × DM + 0.50052 × MGD (R² = 0.6798), where DM is dry matter. Yeast's energy values are influenced by origin and composition, enabling accurate predictions for broiler chickens. Implications: These findings support the use of sugarcane yeast in poultry feeds, enhancing sustainability, reducing industry waste, and improving feed efficiency for global livestock and food production.

## 1 Introduction

The increasing demand for sustainable and cost-effective feed ingredients in poultry production has intensified interest in agro-industrial by-products as alternative feedstuffs. These ingredients offer significant economic advantages due to their low cost and regional availability near processing facilities, making them particularly attractive for intensive poultry operations (Gómez-García et al., 2021). Among various by-products, those of microbial origin, particularly sugarcane yeast (*Saccharomyces cerevisiae*) have emerged as promising alternatives due to their high protein content, essential amino acid profile, and energy density (Alshelmani et al., 2021a; Alshelmani et al., 2021b; Formann et al., 2020).

Sugarcane yeast, generated as a by-product during ethanol fermentation from sugarcane juice, represents both an environmental challenge and an opportunity for the livestock industry. The sugar and alcohol industry produces substantial quantities of this by-product, and its disposal has been restricted due to environmental concerns (Renó et al., 2014). However, the nutritional composition of sugarcane yeast makes it a valuable resource for animal nutrition, potentially transforming an environmental problem into a sustainable feed ingredient (Silva et al., 2014; Santos et al., 2020).

From a nutritional perspective, dehydrated sugarcane yeast functions as a bioactive ingredient, containing B-complex vitamins, digestive enzymes, chelated minerals, and bioactive peptides which enhance animal performance, immune function, and stress resistance (Shruthi et al., 2022). However, like other alternative feedstuffs, yeast nutritional composition varies considerably depending on substrate characteristics, fermentation conditions, and processing methods. Literature reports indicate substantial variation in key nutrients: dry matter (88.0 % - 93.9 %), crude protein (19.4 % - 44.4 %), and ash content (9.8 % -14.4 %), with potassium as the predominant mineral component (NRC, 1994; Rostagno et al., 2005; Chand et al., 2014). Ether extract content typically ranges from 0.9 % to 1.6 %, while apparent metabolizable energy (AME) values for broiler chickens vary from 1963 to 2819 kcal kg^-1^ (Rostagno et al., 2024; Chand et al., 2014).

Determination of metabolizable energy values is crucial for optimal feed formulation in poultry production. Traditional methods involve direct measurement through metabolism trials, which quantify the difference between energy intake and excretion. However, these biological assays are time-consuming, expensive, and impractical for routine feed evaluation (Formann et al., 2020). Alternatively, prediction equations based on chemical composition offer a rapid and cost-effective approach for estimating energy values, enabling nutritionists to assess feed ingredients without conducting extensive animal trials.

Despite the potential of sugarcane yeast as a feed ingredient, limited research has focused on developing accurate prediction equations for metabolizable energy across different broiler growth phases. The variability in yeast composition from different sources and the age-related changes in broiler digestive capacity create additional complexity in energy prediction. Furthermore, existing energy prediction models for yeast have not adequately considered the interaction between chemical composition and bird age, limiting their practical application in commercial feed formulation.

Therefore, this study aimed to determine the metabolizable energy values of sugarcane yeast samples from different origins and develop comprehensive prediction equations based on chemical composition for broiler chickens across production phases. We hypothesized that yeast source and chemical composition significantly influence metabolizable energy values, and that age-specific prediction equations would provide more accurate energy estimates than generalized models. The development of such equations would enhance the precision of feed formulation and promote the sustainable utilization of sugarcane yeast in broiler nutrition.

## 2 Material and methods

### 2.1 Sample collection and chemical analysis

Four sugarcane yeast samples were obtained from different sugar and ethanol production facilities located throughout the Northeast region of Brazil. The samples were transported under appropriate conditions to 2 analytical facilities at the Federal Rural University of Pernambuco.

Chemical composition analyses were conducted to determine dry matter, nitrogen, gross energy, crude protein, ether extract, neutral detergent fiber (NDF), and ash content following standardized methodologies described by AOAC (2012) and Detmann et al. (2025). The mean geometric diameter (MGD) of yeast particles was measured using the protocol established by Banzolli et al. (2015), employing a vibrating sieve system (KUHARDT VIATEST 76773) with 7 sieves of decreasing aperture sizes: 4.0, 2.0, 1.20, 0.60, 0.30, 0.15, and 0.0 mm.

### 2.2 Experimental design and housing

The experiment followed a completely randomized design with 5 dietary treatments and 6 replicates, totaling 30 experimental units. Housing density varied by growth phase: 8 birds per pen during the pre-initial phase (1–8 days), 6 birds during the initial phase (11–18 days), 5 birds during grower phase I (21–28 days), and 4 birds during growth phase II (31–38 days).

The dietary treatments consisted of: (1) a reference diet (RR) formulated with corn and soybean meal as primary ingredients, and (2–5) 4 test diets containing 70 % reference diet plus 30 % of each respective yeast sample. All diets were formulated to meet or exceed nutritional requirements according to Rostagno et al. (2024) recommendations for each production phase (Table 1). Birds had ad libitum access to feed and water throughout the experimental period.

**Table 1:** Composition and nutritional values ​​of basal diets.

Ingredients	Experimental phases, days old			
	1 – 8	11 – 18	21 – 28	31 – 38
Corn	55.41	56.37	60.28	64.52
Soybean meal 45 %	37.50	35.78	31.74	27.70
Soybean oil	2.43	3.680	4.259	4.202
Dicalcium phosphate	1.941	1.835	1.689	1.537
Calcitic limestone	0.921	0.888	0.842	0.801
Common salt	0.517	0.502	0.477	0.452
L-Lysine HCL (78.8 %)	0.374	0.203	0.209	0.264
DL- Methionine (99 %)	0.193	0.083	0.244	0.239
L-Threonine (98.5 %)	0.148	0.058	0.052	0.070
Vitamin-mineral supplement^1^	0.600	0.600	-	-
Vitamin supplement^2^	-	-	0.100	0.100
Mineral supplement^3^	-	-	0.100	0.100
Total	100.0	100.0	100.0	100.0
**Energy and nutritional composition**				
Metabolizable energy, kcal kg^-^1	2960	3050	3150	3200
Dry matter, %	90.25	90.10	91.53	89.70
Crude protein, %	22.11	21.14	19.73	18.31
Calcium, %	0.942	0.899	0.837	0.775
Phosphorus available, %	0.471	0.449	0.418	0.386
Sodium, %	0.224	0.218	0.208	0.198
Potassium, %	0.840	0.812	0.749	0.687
Digestible amino acids, %				
Methionine + cystine	0.968	0.844	0.791	0.755
Methionine	0.677	0.559	0.522	0.500
Lysine	1.363	1.189	1.099	1.048
Threonine	0.886	0.773	0.714	0.681
Tryptophan	0.243	0.234	0.213	0.193
Crude fiber (%)	2.985	2.910	2.759	2.614
Ethereal extract (%)	5.039	6.292	6.887	6.973
Mineral matter (%)	2.913	2.825	2.637	2.453

() Determined values

1 Vitamin-mineral supplement. (Warranty levels per kg of product). Vit. A: 1500.000 UI, Vit. D3: 500.000 UI. Vit. E: 3.333 mg. Vit K3: 250 mg, Riboflavin: 1.000 mg. Thiamine: 300 mg, Vit B6 (Pyrixin): 500 mg, Vit B12: 3.333 mg, Niacin: 6667 mg, Calcium pantothenate: 2.000 mg, Folic acid: 285 mg, Biotin: 8,33 mg, choline chloride: 70 mg, Methionine: 295 mg, Iron: 5000 mg, Copper: 1.550 mg, Manganese: 14.000 mg, Zinc: 12.130 mg, Iodine: 280 mg, Selenium: 70 mg, Robenidine: 6.000 mg, Lincomycin: 734 mg, Antioxidant Additive: 16.667 mg.

2 Vitamin supplement. (Warranty levels per Kg of product). Vit. A: 1000,000 UI, Vit. B3: 2000,000 UI, Vit. E: 20,000 mg, Vit. K3: 4000 mg, Vit. B1: 1880 mg, Vit. B2: 5000 mg, Vit. B6: 2000 mg, Vit. B12: 1000 mg, Niacin: 30,000 mg, Pantothenic acid: 13,500 mg, Folic acid: 500 mg, Selenium: 250 mg, Antioxidant: 100,000 mg.

3 Mineral supplement. (Warranty levels per Kg of product). Manganese: 75,000 mg, Zinc: 70,000 mg, Iron: 60,000 mg, Copper: 85,000 mg, Iodine: 1500 mg, Cobalt: 200 mg.

Environmental conditions were monitored continuously, with recorded average temperatures and relative humidity as follows: pre-initial phase (33.2 °C), initial phase (30.3 °C, 70.9 % RH), growth phase I (30.8 °C, 71.7 % RH), and growth phase II (31.1 °C, 74.8 % RH).

### 2.3 Digestibility trials

Metabolic trials were conducted sequentially in the Non-Ruminant Metabolism Laboratory, Department of Animal Science, UFRPE, corresponding to 4 distinct production phases: pre-initial (1–8 days), initial (11–18 days), growth I (21–28 days), and growth II (31–38 days). A total of 690 male Cobb 500 broiler chickens were utilized across all phases: 240 birds for pre-initial, 180 for initial, 150 for growth I, and 120 for growth II phases.

Birds were housed in individual cages (1.00 × 0.50 × 0.50 m) equipped with trough-type feeders, cup drinkers, and excreta collection trays. Each trial followed an 8-day protocol: 4 days for adaptation to experimental facilities and diets, followed by 4 days of total excreta collection.

Feed intake and excreta production were recorded daily for each experimental unit. Ferric oxide (Fe₂O₃) was incorporated into experimental diets at 2.0 % as a fecal marker to delineate collection periods. Unmarked excreta from the initial collection period and marked excreta from the final collection were discarded to ensure accurate sample representation.

Daily excreta collections were immediately weighed, stored in labeled plastic bags, and frozen at -20 °C. Following completion of each trial, excreta samples were thawed, homogenized by experimental unit, pre-dried in a forced-air oven, ground, and analyzed for dry matter, nitrogen, and gross energy content using methods described by AOAC (2012).

### 2.4 Calculations and statistical analysis

Apparent metabolizable energy (AME) and nitrogen-corrected apparent metabolizable energy (AME_n_) values were calculated using equations proposed by Matterson et al. (1965) and Hill and Anderson (1958). Apparent metabolizability coefficients for dry matter (AMC_DM_) and gross energy (AMC_GE_) were determined using the following formulas:

• Apparent metabolizability coefficient = [(DM ingested - DM excreted) / DM ingested] × 100
• Apparent metabolizability coefficient = (AME_n_ / GE) × 100
• $AME\left(kcalkg^{-1}\right)=\frac{GrossEnergy-GrossEnergyExcreted}{DryMatterIntake}$
• $AME_{n}\left(kcalkg^{-1}\right)=\frac{GrossEnergy-(GrossEnergyExcreted+8.22\cdot NitrogenBalance)}{DryMatterIntake}$

Chemical composition data from yeast samples, including dry matter, crude protein, extract, mineral matter, neutral detergent fiber, gross energy, and mean geometric diameter, were subjected to multiple regression analysis. These parameters served as independent variables, with AME and AME_n_ values as dependent variables. Prediction equations were developed using the Stepwise Backward Elimination method.

Model fit was evaluated using coefficients of determination (R²) and the Akaike Information Criterion (AIC) according to Akaike (1974). All statistical analyses were performed using SAS software version 9.1.3 (SAS Institute Inc, 2008).

## 3 Results

### 3.1 Chemical composition of sugarcane yeast samples

The 4 sugarcane yeast samples showed considerable variability in nutritional composition, reflecting differences in their processing origins (Table 2). Dry matter content was highest in Yeast I and IV (90.71 % and 90.47 %, respectively), while Yeast II and III showed slightly lower values (88.84 % and 89.99 %, respectively). Crude protein levels varied substantially, ranging from 14.94 % in Yeast III to 21.12 % in Yeast IV.

**Table 2:** Physicochemical composition and energy value of the evaluated sugarcane yeasts (expressed in dry matter).

Nutrients	Yeasts			
	I	II	III	IV
Dry Matter, (%)	90.71	88.84	89.99	90.47
Crude Protein, (%)	17.11	18.05	14.94	21.12
Mineral Matter, (%)	7.54	8.05	9.1	9.0
Ethereal Extract, (%)	0.37	0.44	0.49	0.30
NDF 1, (%)	6.86	7.65	3.02	12.13
Gross Energy, (kcal kg^-^1)	4214	4268	4011	4095
MGD^2^, (µm)	661	695	933	616

1 NDF- Neutral detergent fiber.

2 MGD – Mean geometric diameter.

Mineral matter content was elevated in Yeast III and IV (9.1 % and 9.0 %, respectively) compared to Yeast I and II (7.54 % and 8.05 %, respectively). Ether extract remained consistently low across all samples (0.30–0.49 %), with Yeast IV exhibiting the lowest value (0.30 %). Neutral detergent fiber content showed the greatest variation, ranging from 3.02 % in Yeast III to 12.13 % in Yeast IV. Gross energy values ranged from 4011 kcal kg^-1^ (Yeast III) to 4268 kcal kg^-1^ (Yeast II). Mean geometric diameter varied from 616 μm (Yeast IV) to 933 μm (Yeast III).

### 3.2 Energy utilization in experimental diets

The reference diet consistently demonstrated higher energy metabolizability coefficients across all experimental phases compared to yeast-enriched diets, indicating that yeast inclusion reduced overall dietary energy utilization (Table 3).

**Table 3:** Average values ​​of feed intake, apparent metabolizable energy (AME), corrected apparent energy (AMEn) and the apparent metabolizability coefficients of dry matter (AMC_DM_) and gross energy (AMC_GE_) of the diets.

Variables	Diets					Average	*P*-value	SEM
	RD	I	II	III	IV			
**Pre-initial (1 to 8 days)**								
Feed Intake (g)	713.2^e^	819.6^a^	769.7^c^	788.2^b^	732.6^d^	764.6	0.001	1.059
AME (kcal kg^-1)11^)	3,186^a^	2,808^d^	2,888^c^	2,927^b^	2,776^e^	2917	0.001	2.274
AME_n_ (kcal kg^-11^)	3,009^a^	2,618^d^	2,722^c^	2,756^b^	2,618^d^	2745	0.001	2.100
AMC_DM_ (%)	61.63	63.89	64.82	66.52	63.53	64.08	0.213	0.506
AMC_GE_ (%)	66.95^a^	61.53^c^	60.72^d^	63.03^b^	58.58^e^	62.16	0.001	0.048
**Initial (11 to 18 days)**								
Feed Intake (g)	1.690^c^	1.723^b^	1.736^a^	1.715^b^	1.673^d^	1.708	0.001	0.001
AME (kcal kg^-1^)	3,607^a^	3,127^b^	2,960^d^	2,982^d^	3,055^c^	3146	0.001	4.667
AME_n_ (kcal kg^-1^)	3,215^a^	2,804^b^	2,631^c^	2,672^c^	2,757^b^	2816	0.001	4.928
AMC_DM_ (%)	73.41^a^	71.27^b^	70.77^b^	71.74^b^	71.50^b^	71.74	0.001	0.160
AMC_GE_ (%)	69.23^a^	60.68^b^	57.98^d^	59.09^c^	60.22^b^	61.44	0.001	0.106
**Grower I (21 to 28 days)**								
Feed Intake (g)	2,257^c^	2,406^b^	2,451^a^	2,458^a^	2,418^b^	2398	0.001	1.795
AME (kcal kg^-1^)	3,671^a^	2,950^b^	2,924^cd^	2,920^d^	2,943^bc^	3081	0.001	2.419
AME_n_ (kcal kg^-1^)	3,224^a^	2,617^b^	2,566^c^	2,544^c^	2,606^b^	2711	0.001	2.821
AMC_DM_ (%)	79.14^a^	73.58^c^	74.02^c^	78.66^ab^	75.91^bc^	76.26	0.001	0.295
AMC_GE_ (%)	69.47^a^	57.37^c^	56.91^c^	56.30^c^	58.17^b^	59.64	0.001	0.062
**Grower II (31 to 38 days)**								
Feed Intake (g)	1.909^e^	2.167^a^	2.099^b^	2.072^c^	1.984^d^	2.046	0.001	1.336
AME (kcal kg^-1^)	3,404^a^	2,813^c^	2,696^d^	2,871^b^	2,693^d^	2895	0.001	2.009
AME_n_ (kcal kg^-1^)	3,356^a^	2,770^c^	2,657^d^	2,832^b^	2,658^d^	2855	0.001	2.04
AMC_DM_ (%)	84.92^a^	84.28^ab^	83.03^ab^	82.03^ab^	81.05^b^	83.06	0.030	0.443
AMC_GE_ (%)	66.95^a^	61.53^c^	60.72^d^	63.03^b^	58.58^e^	62.16	0.001	0.067

*P* = probability; CV = coefficient of variation.

RD- Reference diets; I- RD + 30 % of Yeast 2; II - RD + 30 % of Yeast 1; III- RD + 30 % of Yeast 3; IV - RD + 30 % of Yeast 4.

^A, b^ Within a row, values with different letters differ significantly from the source by Tukey’s test (*P* < 0.05).

**Pre-initial phase (1–8 days):** Feed intake was highest (*P* = 0.01) with Yeast I (819.6 g) and lowest with Yeast IV (732.6 g). Birds fed diets containing Yeast III achieved the highest (*P* = 0.01) AME and AMEn values (2927 and 2756 kcal kg^-1^, respectively), followed by Yeast II (2888 and 2722 kcal kg^-1^). Yeast IV shows the lowest (*P* = 0.01) energy values (2776 and 2618 kcal kg^-1^ for AME and AMEn, respectively). The apparent gross energy metabolizability coefficient was superior (*P* = 0.01) for Yeast III (63.03 %) compared to other samples, however the DM apparent coefficient was not different between groups (*P* = 0.21).

**Initial phase (11–18 days):** Feed intake was greatest (*P* = 0.01) with Yeast II (1736 g) and lowest with Yeast IV (1673 g). Yeast I demonstrated the highest (*P* = 0.01) AME value (3127 kcal kg^-1^), while Yeast II and III showed lower means (2960 and 2982 kcal kg^-1^, respectively). For AMEn, Yeast I and IV achieved comparable (*P* = 0.01) results (2804 and 2757 kcal kg^-1^). The AMC_DM_ was similar between Yest, however, the Yest I and IV showed the highest values for AMC_GE_ (*P* = 0.01).

**Growth phase I (21–28 days):** Yeast I maintained the highest (*P* = 0.01) AME value (2950 kcal kg^-1^), while other samples showed similar performance (2920–2943 kcal kg^-1^). For AMEn, Yeast I and IV performed (*P* = 0.01) comparably (2617 and 2606 kcal kg^-1^). Feed intake was highest (*P* = 0.01) with Yeast II and III (2458 and 2451 g, respectively). The AMC_DM_ was similar between Yest III and IV, but birds fed Yest IV had the highest values for AMC_GE_ (*P* = 0.01).

**Growth phase II (31–38 days):** Birds fed Yest I had (*P* = 0.01) the highest Feed intake between treatments. Yeast III achieved the highest (*P* = 0.01) energy values (AME: 2871 kcal kg^-1^; AMEn: 2832 kcal kg^-1^), followed by Yeast I (AME: 2813 kcal kg^-1^; AMEn: 2770 kcal kg^-1^). Yeast II and IV showed similar, lower performance for both variables. The AMC_DM_ was similar between Yest I, II, and III with the higher means (*P* = 0.03), but the Yest III showed the highest value for AMC_GE_ (*P* = 0.01).

### 3.3 Energy values of yeast samples

According to the results in Table 4, when evaluated independently, the yeast samples demonstrated significant variation in metabolizable energy content across the different growth phases. During the pre-initial phase (1 to 8 days), the AME ranged from 2223 kcal kg^-1^ for Yeast III to a low value of 1537 kcal kg^-1^for Yeast IV (*P* < 0.05). In Yeast I and Yeast III the data also shows a trend where energy values decreased as birds aged. On the other hand, other yeasts showed different patterns (*P* < 0.05), as observed in Yeast II the AME value decreased through the first 3 phases but then increased in the final grower phase, suggesting age-related interactions between yeast composition and digestive capacity.

**Table 4:** Average values of apparent metabolizable energy (AME), corrected apparent (AMEn) of sugarcane yeasts and the apparent metabolizability coefficients of dry matter (AMC_DM_) and gross energy (AMC_GE_).

Variables	Yeasts				Average	*P*-value	CV
	I	II	III	IV			
**Pre-initial (1 to 8 days)**							
AME (kcal kg^-^1)	2,090^b^	1,764^c^	2,223^a^	1,674^d^	1937	0.001	10.438
AME_n_ (kcal kg^-^1)	1,953^b^	1,537^c^	2,069^a^	1,566^c^	1781	0.001	10.179
AMC_DM_ (%)	73.37	70.12	79.81	73.15	73.15	0.389	3.912
AMC_GE_ (%)	46.35^b^	36.03^d^	51.60^a^	38.24^c^	43.06	0.001	0.248
**Initial (11 to 18 days)**							
AME (kcal kg^-^1)	1,229^c^	1,802^a^	1,285^c^	1,571^b^	1472	0.001	25.24
AME_n_ (kcal kg^-^1)	1,070^d^	1,670^a^	1,197^c^	1,527^b^	1366	0.001	28.664
AMC_DM_ (%)	63.71	65.37	67.20	68.37	66.16	0.160	1.48
AMC_GE_ (%)	25.41^c^	39.13^a^	29.84^b^	37.30^a^	32.92	0.001	0.689
**Grower I (21 to 28 days)**							
AME (kcal kg^-^1)	924^ab^	961^a^	879^b^	986^a^	938	0.028	18.19
AMEn (kcal kg^-^1)	803^b^	940^a^	698^c^	944^a^	846	0.001	23.382
AMC_DM_ (%)	60.32^b^	58.25^b^	77.33^a^	67.63^ab^	65.88	0.001	2.725
AMC_GE_ (%)	19.07^b^	22.04^a^	17.40^b^	23.06^a^	20.39	0.001	0.561
**Grower II (31 to 38 days)**							
AME (kcal kg^-^1)	800^c^	1,181^b^	1,422^a^	779^c^	1045	0.001	12.244
AME_n_ (kcal kg^-^1)	785^c^	1,154^b^	1,408^a^	781^c^	1032	0.001	12.302
AMC_DM_ (%)	77.98^ab^	82.52^a^	74.18^ab^	68.55^b^	75.81	0.004	2.392
AMC_GE_ (%)	18.64^c^	27.04^b^	35.11^a^	19.08^c^	24.97	0.001	0.296

*P* = probability; CV = coefficient of variation.

1 – Yeast Distillery 1 (Diet C); 2 – Yeast Distillery 2 (Diet B); 3 – Yeast Distillery 3 (Diet D); 4 – Yeast distillery 4 (Diet 5). ^A, b^ Within a row, values with different letters differ significantly from the source by Tukey’s test (*P* < 0.05).

### 3.4 Prediction equations for energy values

Multiple regression analysis generated several prediction models for estimating AME and AMEn values based on chemical composition (Table 5, Table 6, Table 7, Table 8). The AME Prediction Models**:** The most robust equations without intercepts achieved R² values exceeding 0.96, with the best-fitting model being:

$$AME=-32.13\left(age\right)+49.37\left(CP\right)+2736.4\left(EE\right)\left[R^{2}=0.97\right]$$

**Table 5:** Equations for predicting AME (kcal kg^-1^) of sugarcane yeast for broilers based on its physical-chemical composition1.

RE	Regression coefficient									R^2^	AIC
	Intercept	Age	DM	CP	MM	EE	NDF	MGD	GE		
**1**	8905.51	-32.1006	-81.048	—	—	—	—	0.50052	—	0.680	1071.3604
**2**	-2666.26	-32.1006	—	136.940	—	5496.06	—	—	—	0.680	1071.3926
**3**	968.43	-32.1006	—	—	—	2104.38	22.159	—	—	0.679	1071.5403
**4**	—	-32.1253	—	49.372	—	2736.44	—	—	—	0.968	1070.0169
**5**	—	-32.1032	41.444	-123.228	—	—	59.644	—	—	0.968	1071.4826
**6**	—	-32.1032	9.545	—	—	2308.82	25.886	—	—	0.968	1071.4875
**7**	—	-32.1032	—	36.863	—	2999.92	15.797	—	—	0.968	1071.4889
**8**	—	-32.1033	-14.902	94.450	—	4078.07	—	—	—	0.968	1071.4913
**9**	—	-32.1195	—	-39.032	90.297	—	—	—	0.46072	0.968	1071.5986
**10**	—	-32.1550	—	—	-667.258	—	247.075	7.9364	—	0.968	1071.6804

1 – Values ​​based on dry matter. RE - Regression equation; DM - Dry matter CP – Crude protein; MM – Mineral matter; EE – Ethereal extract; NDF – Neutral detergent fiber; MGD – Mean geometric diameter; GE – Gross energy; R^2^ – Coefficient of determination; AIC- Akaike information criterion.

**Table 6:** Prediction equations for AME_n_ (kcal kg^-1^) of sugarcane yeast for broilers based on physical-chemical composition1.

RE	Regression coefficients									R^2^	AIC
	Intercept	Age	DM	CP	MM	EE	NDF	MGD	GE		
**1**	-4190.27	-27.6797	—	184.997	—	6718.77	—	—	—	0.576	1085.744
**2**	14,361.06	-27.6797	-115.544	—	—	—	—	—	-0.5221	0.576	1085.774
**3**	8198.04	-27.6797	-74.273	—	—	—	—	0.3902	—	0.575	1085.892
**4**	—	-27.7557	—	—	-519.001	—	202.870	6.4243	—	0.957	1086.003
**5**	—	-27.7185	—	47.377	—	2381.78	—	—	—	0.957	1085.067
**6**	—	-27.6858	38.318	-119.241	—	—	64.030	—	—	0.957	1086.056
**7**	—	-27.6857	7.456	—	—	2233.32	31.348	—	—	0.957	1086.066
**8**	—	-27.6856	—	28.785	—	2773.38	23.479	—	—	0.957	1086.068
**9**	—	-27.7120	—	-29.099	95.567	—	—	—	0.3645	0.957	1086.68
**10**	—	-27.5216	—	—	—	2298.95	55.062	0.6399	—	0.957	1086.070

1 – Values based on dry matter. RE - Regression equation; DM - Dry matter CP – Crude protein; MM – Mineral matter; EE – Ethereal extract; NDF – Neutral detergent fiber; MGD – Mean geometric diameter; GE – Gross energy; R^2^ – Coefficient of determination; AIC- Akaike information criterion.

**Table 7:** Estimation of AME values of sugarcane yeasts using prediction equations, based on dry matter.

Equations with intercept											
AME _1_ = 8905.51 - 32.1006(age) - 81.048(DM) + 0.50052(MGD) R^2^ = 0.679											
AME _2_ = - 26,660.26 - 32.1006(age) + 136.940(CP) + 5496.06(EE) R^2^ = 0.679											
AME _3_ = 968.43 - 32.1006(age) + 2104.38(EE) + 22.159(NDF) R^2^ = 0.679											

Equations without intercept											
AME _4_ = - 32.1253(age) + 49.372(CP) + 2736.44(EE) R^2^ =0.968											
AME _5_ = - 32.1032(age) + 41.444(DM) - 123.228(CP) + 59.644(NDF) R^2^ = 0.968											
AME_6_ = - 32.1032(age) + 9.545(DM) + 2308.82(EE) + 25.886(NDF) R^2^ = 0.968											
AME _7_ = - 32.1032(age) + 36.863(CP) + 2999.92(EE) + 15.797(NDF) R^2^ = 0.968											
AME _8_ = -32.1033(age) -14.902(DM) + 94.450(CP) + 4078.07(EE) R^2^ = 0.968											
AME _9_ = - 32.1195(age) - 39.032(CP) + 90.297(MM) + 0.46072(GE) R^2^ = 0.968											
AME_10_ = - 32.1551(age) - 667.258(MM) + 247.075(NDF) + 7.9364(MGD) R^2^ = 0.968											

Feed	AME^1^	AME_1_^2^	AME_2_^2^	AME_3_^2^	AME_4_^2^	AME_5_^2^	AME_6_^2^	AME_7_^2^	AME_8_^2^	AME_9_^2^	AME_10_^2^
Yeast 1	1261.10	1219.34	1265.12	1273.1	1295.96	1271.44	1271.66	1271.71	1271.82	1276.62	1282.74
Yeast 2	1427.60	1388.73	1433.48	1437.91	1409.51	1435.76	1435.87	1435.94	1435.92	1409.22	1407.47
Yeast 3	1452.50	1414.15	1446.73	1440.53	1452.03	1442.63	1442.44	1442.39	1442.3	1460.2	1451.75
Yeast 4	1252.80	1216.34	1248.77	1242.57	1237.22	1244.33	1244.16	1244.13	1244.01	1248.63	1253.5
Average	1348.5	1309.64	1348.5	1348.5	1348.7	1348.5	1348.5	1348.5	1348.5	1348.7	1348.9
SSD^3^	—	537,625.3	537,625.3	537,625.3	538,453	537,712.4	537,712.4	537,712.4	537,715.8	528,258.5	539,449.1
Average SSD^4^	—	134,406.3	134,406.3	134,406.3	134,613.2	134,428.1	134,428.1	134,428.1	134,428.9	134,564.6	134,862.2

1- AME- apparent metabolizable energy, observed “in vivo” in tests with chickens.

2 - AME estimates by prediction equations.

3 - Sum of squared deviations.

4 - Average of the sum of squares of deviations.

**Table 8:** Estimation of AME_n_ values of sugarcane yeasts using prediction equations, based on dry matter.

Equations with intercept											
AME_n 1_ = - 4190.27 - 27.6797(age) + 184.997(CP) + 6718.77(EE) R^2^ = 0.576											
AME_n 2_ = 14,361.06 – 27.6797(age)115.544(DM) - 0.5221(GE) R^2^ = 0.576											
AME_n 3_ = 8198.04 - 27.6797(age) - 74.273(DM) + 0.3902(MGD) R^2^ = 0.575											

Equations without intercept											
AME_n 4_ = - 27.7557(age) - 519.001(MM) + 202.870(NDF) + 6.4243(MGD) R^2^ =0.957											
AME_n 5_ = - 27.7557(age) + 47.377(CP) + 2381.78(EE) R^2^ = 0.957											
AME_n 6_ = - 27.6858(age) + 38.318(DM) - 119.241(CP) + 64.030(NDF) R^2^ = 0.957											
AME_n 7_ = - 27.6857(age) + 7.456(DM) + 2233.32(EE) + 31.348(NDF) R^2^ = 0.957											
AME_n 8_ = - 27.6856(age) + 28.785(CP) + 2773.38(EE) + 23.479(NDF) R^2^ = 0.957											
AME_n 9_ = - 27.7120(age) - 29.099(CP) + 95.567(MM) + 0.3645(GE) R^2^ = 0.957											
AME_n 10_ = - 27.5216(age) + 2298.95(EE) + 55.062(NDF) + 0.6399(MGD) R^2^ = 0.957											

Feed	AME_n_^1^	AME_n1_^2^	AME_n2_^2^	AME_n3_^2^	AME_n4_^2^	AME_n5_^2^	AME_n6_^2^	AME_n7_^2^	AME_n8_^2^	AME_n9_^2^	AME_n10_^2^
Yeast 1	1153.30	1165.41	1140.18	1178.90	1183.65	1213.18	1177.59	1177.89	1177.85	1179.9	1114.64
Yeast 2	1325.80	1344.3	1328.05	1331.06	1297.65	1305.05	1344.92	1344.99	1345.06	1294.29	1340.82
Yeast 3	1326.03	1326.03	1329.35	1338.51	1342.39	1333.65	1320.27	1320.09	1322.04	1356.55	1353.13
Yeast 4	1205.0	1192.74	1230.04	1179.17	1205.94	1173.9	1185.07	1184.92	1184.88	1197.78	1215.09
Average	1252.5	1257.1	1256.9	1256.9	1257.4	1256.4	1256.9	1257.4	1257.4	1257.1	1255.9
SSD^3^	—	399,738.7	399,738.7	399,738.7	401,936.8	401,936.8	399,914.9	399,912	399,908.1	400,672.1	395,185.3
Average SSD^4^	—	99,934.6	99,934.6	99,934.6	100,484.2	100,484.2	99,978.7	99,978	99,977.2	100,168	98,796.3

1- AME- apparent metabolizable energy, observed “in vivo” in tests with chickens;.

2 - AME estimates by prediction equations;.

3 - Sum of squared deviations;.

4 - Average of the sum of squares of deviation.

Equations incorporating intercepts showed lower predictive accuracy (R² = 0.68), with the primary model:

$$AME=8905.51-32.10\left(age\right)-81.05\left(DM\right)+0.50\left(MGD\right)\left[R^{2}=0.68\right]$$

AME_n_ prediction models: Similar patterns emerged for AMEn predictions, with intercept-free equations demonstrating superior fit (R² > 0.95). The optimal model incorporated multiple variables:

$$AME_{n}=-27.76\left(\text{age}\right)-519.0\left(MM\right)+202.9\left(NDF\right)+6.42\left(MGD\right)\left[R^{2}=0.96\right]$$

Age consistently emerged as a significant negative predictor across all models, reflecting the observed decline in energy utilization with advancing bird age. Chemical composition variables, particularly crude protein and ether extract showed positive associations with energy values.

### 3.5 Model validation and accuracy

Comparison between observed and predicted energy values revealed close agreement for most equations (Table 7, Table 8). Models without intercepts demonstrated superior predictive accuracy, with average sum of squared deviations ranging from 134,406 to 134,862 for AME predictions and 98,796 to 100,484 for AME_n_ predictions.

## 4 Discussion

The physicochemical composition of the different sugarcane yeast samples exhibited considerable variation. Such variations demonstrate that the chemical and energetic composition of feed ingredients can be influenced by multiple factors, including soil conditions, climate, and genetic variability of the source material. For by-products such as sugarcane yeast, processing duration and inadequate storage conditions can further alter the final composition (Freitas et al., 2013).

The DM values obtained for yeasts I, II, and III in the present study align with literature values, falling within the range of 89.1 to 93.87 % reported by Battisti et al. (1985); Generoso et al. (2008); Butolo (1997), and Longo et al. (2005). However, yeast II exhibited a lower value (88.84 %). The highest crude protein content (21.12 %) was observed in yeast IV, nevertheless, this value was lower than those reported by Silva (2010), Rostagno et al. (2024), Yamada et al. (2003), and Faria et al. (2000), which ranged from 30.35 to 39.6 % when evaluating spray-dried yeasts. Conversely, our values were higher than 14.10 % reported by Lopes (2010), which is similar to our findings for yeast III.

The yeast samples presented NDF values ranging from 3.02 to 12.13 %. These values exceeded those reported by Generoso et al. (2008) 1.00–1.09 %, but were lower than those presented by Silva (2007), who evaluated agro-industrial by-products for poultry feed and obtained NDF concentrations between 49.87 % and 57.28 % in sugarcane yeasts. The GE values of the evaluated yeasts ranged from 4011 to 4268 kcal kg^-1^, with yeasts I, II, and IV showing characteristics similar to those described by Generoso et al. (2008), Rostagno et al. (2024), and Longo et al. (2005) (4255, 4157, and 4077 kcal kg^-1^, respectively). Only yeast III exhibited a lower GE content (4011 kcal kg^-1^).

The observed variability in chemical composition among the evaluated samples can be attributed to the processing methods employed by different industries to obtain these by-products. According to Perdomo et al. (2004), yeast chemical composition and nutritional value are influenced by substrate origin and industrial processing conditions. Understanding the chemical composition of ingredients in broiler diets is crucial for determining nutrient digestibility percentages, which directly affects production efficiency.

Feed intake of experimental diets enriched with different yeast samples increased during the pre-initial, initial, and grower I phases, however, it decreased considerably during growth phase II. Given the elevated ambient temperatures during this experimental phase, this reduction in consumption was likely attributed to environmental factors. The interactions among the environment, animal and diet are regulated by the amount of heat that the animal can dissipate into the environment (Emmans 1987). The actual feed intake is the capacity of the animal to consume under the influence of environmental limitation of the gastrointestinal tract and type of diet (Santos et al., 2016)

During the pre-initial and initial experimental phases, energy utilization was considerably higher than in growth phases I and II, regardless of yeast origin. Santos et al. (2023) reported that sugarcane yeast contains mannan oligosaccharides (MOS) in its cell wall that benefit animal development by adhering to pathogenic bacterial fimbriae, preventing colonization of the digestive tract. Therefore, MOS in poultry diets can stimulate the growth of beneficial intestinal bacteria. The primary mechanism of S. cerevisiae cell wall involves d-mannose, which mediates beneficial effects by improving intestinal lumen health and increasing intestinal absorption area (Freitas et al., 2013). Consequently, nutrient breakdown becomes more efficient, allowing animals to extract more energy and protein from the diet (Santos et al., 2023).

The low AME and AMEn values observed in growth phase I with sugarcane yeast inclusion may be related to limitations in intestinal maturation in the presence of nucleotides. Esteve-García et al. (2007) demonstrated that during the growth period, nucleic acid availability can limit tissue maturation in organs with rapid cellular replication and low biosynthetic capacity, such as the intestine, thereby restricting growth rate due to reduced digestion and nutrient absorption capacity.

Longo et al. (2005) reported that differences in calculated AMEn values, as well as energetic utilization (CMGE) of ingredients, are primarily related to gastrointestinal tract immaturity, which promotes changes in digestive and absorptive capacity. This demonstrates that metabolic characteristics specific to each developmental stage can affect food energy values and consequently alter the metabolizable energy provided in the diet.

Literature reporting gross energy metabolizability coefficients in birds is limited; typically, gross energy and AMEn data are presented separately. However, Faria et al. (2000), studying spray-dried yeast, found digestibility coefficients of 69.60 % and 87.19 % for growing rabbits, while Zanutto et al. (2008) reported a coefficient of 82.94 % for piglets in the initial phase. Silva (2010), working with 30 % replacement of reference feed with spray-dried sugarcane yeast in laying hens, found metabolizability values of 45.18 %. Multiple factors can influence food energy assessment results, with bird age, food processing, and experimental procedures being among the most important (Sakomura & Rostagno, 2016). Bird age is one of the most frequently cited factors causing variations in food metabolizable energy values (Rodríguez-Ortega et al., 2022).

Yeast III samples achieved higher AME and AMEn values than other samples during the pre-initial phase and growth phase II (31 to 38 days of age), as well as higher CMGE values. The superior AME and AME_n_ values of yeast III may be related to its chemical composition, particularly its low NDF content, which may be a limiting factor for energy utilization. Longo et al. (2005), working with 20 % replacement of reference diet with dry yeast in the pre-initial period, obtained an AMEn value of 2037 kcal kg^-1^ in natural matter and 49.97 % for CMGE. The NRC (1994); Rostagno et al. (2024), and D’Agostini et al. (2004) reported AME_n_ values ranging from 1990 to 2536 kcal kg^-1^.

In this trial, AME values were, on average, 8.19 % higher than AME_n_ values, meaning AME values exceeded AME_n_ values by 156.5 kcal kg^-1^. This characteristic is typical when ME values are determined in growing birds, as greater nitrogen retention occurs at this stage for protein tissue deposition (Nery et al., 2007). The birds generally exhibited positive nitrogen balance, characterized by nitrogen retention from feed. Leeson and Summers (2001) stated that estimated energy values must be corrected for nitrogen balance, as it is impossible to ensure uniform growth rates among all birds during metabolism assays. When corrected for nitrogen balance, ME values consistently decrease when birds maintain positive nitrogen balance (Musigwa et al., 2020), explaining the lower AME_n_ values observed in this study. The variations in AME values observed across different growth phases may be attributed to changes in intestinal maturity and absorptive capacity. Nutritional factors that promote favorable changes in intestinal morphology, stimulating increased cell proliferation and reduced apoptosis, thereby enhance the absorptive function of the gastrointestinal tract, and subsequently improve animal performance (Santos et al., 2024). The higher AME values recorded for the sugarcane yeast ingredient during the initial and growth phases compared to the pre-initial phase suggest improved intestinal development and enhanced capacity for nutrient absorption as birds mature.

Predicted AME and AME_n_ values were estimated using regression equations derived from the physicochemical composition of evaluated yeast samples, with and without intercepts. The generated equations closely approximated values determined in biological tests, as evidenced by similar sums of mean squares of deviations across all results. However, model selection for ME prediction will depend on available laboratory analyses, as some analyses require sophisticated equipment that may increase costs.

The age variable was incorporated into all AME and AME_n_ prediction models, highlighting the importance of this factor when considering age effects on predicted values. Given that AME and AME_n_ values of sugarcane yeast decrease by an average of 32.16 and 27.68 kcal kg^-1^, respectively, with each day of broiler chicken age, age represents a critical consideration in energy value predictions.

Equations adjusted using intercepts in the present study exhibited lower coefficients of determination compared to equations without intercepts. Janssen (1989), when developing the European Table of Energy Values of Poultry Foods, excluded intercepts, as did Rostagno et al. (2024) in the Brazilian Poultry and Swine Tables. Data adjustment to models was evaluated using the AIC, in which, the model with the lowest AIC value among competing models is considered optimal.

The coefficients of determination of regression equations ranged from 0.68 to 0.97 for AME and from 0.57 to 0.96 for AMEn, using equations with 3 to 4 variables. Prediction equations enable reduction in the number of required laboratory analyses, consistent with Nunes (1999), who reported that equations with 2 to 4 variables are more practical because they require fewer laboratory analyses, thereby saving time, money, and resources. Equations with 2 to 4 variables provide better estimates of food energy values. However, not all equations with this variable range produce reliable estimates because, despite variable inclusion in the equation, variables must be highly correlated with energy values (Rodrigues et al., 2002).

Although the generated equations fit the observed data well, this study alone cannot accurately predict energy values for all types of sugarcane yeast available commercially. Given the potential benefits that yeast can provide for poultry production, additional studies regarding processing methods, composition, and application in poultry feeds must be conducted to clarify specific characteristics and optimize utilization strategies.

## 5 Conclusion

The industrial origin of the substrate influenced the physicochemical and energetic composition of the yeasts. During the pre-initial phase, Yeast III exhibited higher values for AME, AMEn, and CMDM. In the initial period, Yeast II showed the highest values for these parameters. However, the AME and AMEn values predicted by the generated models closely matched the results obtained from the metabolism tests.

## Declaration of funding

This study was supported by the Coordination for the Improvement of Higher Education Personnel (CAPES, Finance Code 001) and by Karolinska Institutet through an institutional publication agreement. The funding covered the open access publication fees.

## Ethical statement

The study “**Chemical composition, determination, and prediction of energy values of different sugarcane yeasts for broiler chickens**” was approved by Committee of Ethics in Animal Use of Federal Rural University of Pernambuco (License n°. 0782.014).

## CRediT authorship contribution statement

**Emanuela Nataly Ribeiro Barbosa:** Writing – original draft, Methodology, Investigation, Formal analysis, Conceptualization. **Carlos Bôa-Viagem Rabello:** Writing – review & editing, Visualization, Methodology, Investigation. **Marcos Jose Batista dos Santos:** Writing – review & editing, Resources, Methodology, Investigation. **Claudia Costa Lopes:** Writing – review & editing, Software, Methodology, Investigation. **Edney Pereira Silva:** Writing – review & editing, Resources, Methodology, Investigation, Formal analysis. **Júlio Cezar dos Santos Nascimento:** Writing – review & editing, Methodology, Investigation, Formal analysis, Data curation. **Camilla Mendonça Silva:** Writing – review & editing, Visualization, Methodology, Investigation, Formal analysis. **Lilian Francisco Arantes de Souza:** Writing – review & editing, Visualization, Validation, Supervision, Formal analysis. **Lucas Rannier Ribeiro Antonino Carvalho:** Writing – review & editing, Writing – original draft, Resources, Project administration, Funding acquisition, Formal analysis. **Maria do Carmo Mohaupt Marques Ludke:** Writing – review & editing, Writing – original draft, Visualization, Supervision, Resources, Project administration, Investigation, Funding acquisition.

## Declaration of competing interest

The authors declare that they have no known competing financial interests or personal relationships that could have appeared to influence the work reported in this paper.
